# Enhancement of the antibacterial potential of plantaricin by incorporation into silver nanoparticles

**DOI:** 10.1186/s43141-020-00093-z

**Published:** 2021-01-20

**Authors:** Sara Adel Amer, Hala Mohamed Abushady, Rasha Mohamed Refay, Mahmoud Ahmed Mailam

**Affiliations:** 1 Agricultural Research Centre (ARC), Food Technology Research Institute (FTRI), Giza, Egypt; 2 Faculty of Science, Department of Microbiology, Ain Shams University, Cairo, Egypt; 3 Health Affair, Luxor Bacteriological Laboratory, Luxor, Egypt

**Keywords:** Bacteriocin gene, Lactic acid bacteria, Plantaricin EF, Species-specific primer, Nano technology, Bacteriocin-nano silver

## Abstract

**Background:**

Bacteriocins are proteinaceous compounds produced from lactic acid bacteria. Bacteriocins are well-known for their antibacterial potential and safety for application in food. However, the commercial availability of bacteriocin is facing several limitations; among them is the low yield and short stability period. That calls for a new strategy for overcoming these hurdles. Among these approaches is incorporating bacteriocin in nanoparticles. So, the aim of this study was to enhance the plantaricin produced from isolated *Lactobacillus plantarum* strain using nanotechnology.

**Results:**

In this study, the *pln*EF genes encoding plantaricin EF have been identified and sequenced (accession number of MN172264.1). The extracted bacteriocin (EX-PL) was obtained by the ammonium sulfate method. Then, it was used for biosynthesizing plantaricin-incorporated silver nanoparticles (PL-SNPs). The synthesized nanoparticles were confirmed by SEM-EDAX analysis. The antibacterial activity of both combined (PL-SNPs) and extracted plantaricin (EX-PL) were tested against some strains of foodborne pathogenic bacteria. The results revealed that the antibacterial activities were increased by 99.2% on the combination of bacteriocin with the silver nanoparticle. The MIC of EX-PL (7.6 mg/mL) has been lowered after incorporating into silver nanoparticles and reached 0.004 mg/mL for PL-SNPs. Despite that extracted plantaricin showed no inhibitory activity towards *Listeria monocytogenes*, plantaricin-incorporated silver nanoparticles displayed inhibitory activity against this strain. Furthermore, the stability period at 4 °C was increased from 5 days to 60 days for EX-PL and PL-SNPs, respectively.

**Conclusions:**

Plantaricin-incorporated silver nanoparticles possess higher antibacterial activity and more stability than the free one, which makes it more fitting for combating foodborne pathogens and open more fields for applications in both food and pharmaceutical industries.

**Graphical abstract:**

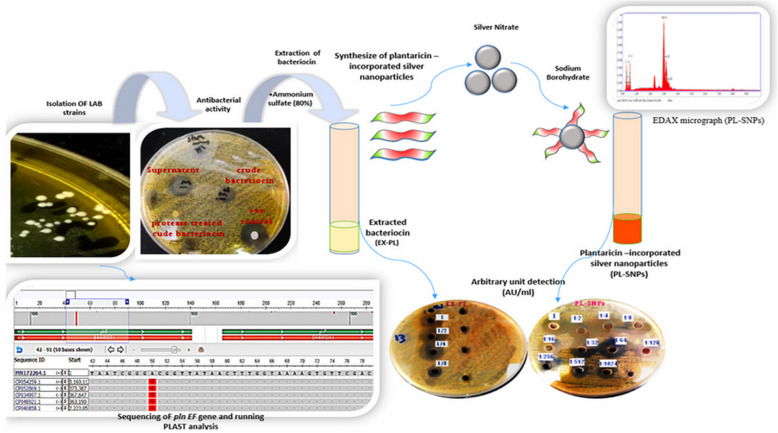

**Supplementary Information:**

The online version contains supplementary material available at 10.1186/s43141-020-00093-z.

## Background

*Lactobacillus* (*Lb.*) *plantarum* is one of the common lactobacilli, frequently found in a diversity of ecological environments [1]. *Lb. plantarum* is “generally recognized as safe” (GRAS) and possesses a qualified presumption of safety (QPS) status, thus facilitate its utilization in the food industry [2]. In fact, *Lb. plantarum* species have been uniquely equipped with a natural ability to produce extracellular bacteriocins like plantaricin (Pln) EF, pln 163, pln Y, and pln JLA-9 [3–6]. One of the most attractive and bioactive bacteriocin metabolites produced by *Lb. plantarum* is the two-peptide bacteriocin plantaricin EF (PlnEF), which consists of two non-identical peptides that are encoded by two separate genes [7–9]. The bactericidal effect of PlnEF is through attacking the target membrane, causing leakage of intracellular compounds and consequently cell death [10, 11].

Nevertheless, until now, bacteriocins application as food preservatives is undergoing various limitations such as degradation by some proteolytic enzymes, limited antimicrobial spectrum, low production and at the same time needing for a high effective dosage [12].

The past decade has seen a rabid development in nanotechnology in many fields of applications such as in medicine, agriculture, cosmetic, electronic and pharmaceutical industries [13]. Nanoparticles may be classified as organic or inorganic based on the components that construct the structure. The organic nanoparticles are biodegradable such as that prepared from a variety of natural polymers; like alginate [14] and chitosan [15, 16]. While many inorganic nanoparticles possess a smaller particle size, improved stability and high drug loadings. Additionally, it has an adaptable potential for different peptide delivery formulations, because of the properties provided by their high surface area. Examples for inorganic nanoparticles include zinc, copper, titanium, and silver [17]. Some of metal nanoparticles have proved to diminish the risks associated with foodborne pathogens [17–19]. Among these, silver nanoparticles have gained a considerable attention because of its antimicrobial properties against many infectious pathogens like *Pseudomonas aeruginosa*, *Escherichia coli*, *Proteus mirabilis*, *Shigella flexaneri*, *Salmonella Somenei*, and *Klebsiella pneumonia*, *Enterococcus faecalis Pseudomonas fluorescens*, *Aeromonas hydrophila*, and *Flavobacterium branchiophilum* [20], consequently, silver nanoparticles incorporated a lot of science fields including foods, cosmetics, and as antimicrobial coatings on the surface of medical devices [21].

Bacteriocin-nanoconjugate is considered one of the newest and encouraging solutions which recently developed to overcome some of the aforementioned defects which hinder bacteriocin- industrial applications. Hence, several studies have been evolved for exploring different nanoformulations for bacteriocin [12]. Bacteriocin incorporated nanoparticles have found to induce several structure changes to bacterial cells that enhancing the interaction with bacterial membrane constituents through disturbing permeability and forming pits, facilitating penetration inside the cell, unbalancing respiratory functions, causing an increase of reactive oxygen species and depletion of ATP levels, intercalating between DNA bases and interfering replication [22, 23]. At present, the most widely investigated bacteriocin nanoconjugated types were with metallic nanoparticles, especially with silver which attracted significant attention. As nanosilver particles proved to be more appropriate for conjugation with bacteriocins, because it provide higher antibacterial potential with broad spectrum against pathogenic microorganisms, more active surface, and greater chemical stability of the nanoparticles that led to increase in released free bacteriocin when exposed to the human gut system without being affected by the digestive enzymes [21, 23].

Because of nisin availability as a commercial form, a considerable amount of literature has been published on nisin-loaded nanoparticles [24, 25]. Although plantaricin have proved to be efficient antibacterial agent [7, 9], there are still insufficient data about plantaricin-loaded nanoparticles. This study, therefore, is set out for sequencing the gene encoding plantaricin EF in the previously isolated *Lb. plantarum s*train, to develop a plantaricin-loaded silver nanoparticles and finally comparing its antibacterial activity with the free one.

## Methods

### Bacterial growth and conditions

Potential antibacterial producer *Lb. plantarum*, strain no. 13 were previously isolated from Egyptian traditional butter, identified by species-specific PCR *rec*A primer and the presence of the gene encoding plantaricin EF (*Pln*EF gene) using specific primer (Table 1) was confirmed as formerly described [28].

**Table 1:** Specific oligonucleotide primers sequences (Metabion; Germany)

Primer	Target gene	Primer sequence	Amplified product	Reference
*Lb. plantarum*	*recA* gene	F: 5 CAGAATTGAGCTGGTGGTGG3- R: 5-TGTTACTTTCGCAACCAGAT3	210 bp	[26]
Bacteriocin gene	*Pln EF*	F: GGCATAGTTAAAATTCCCCCC R:CAGGTTGCCGCAAAAAAAG	428 bp	[27]

*Lb. plantarum,* strain no. 13, was routinely cultivated in MRS broth (HiMedia, Mumbai) at 30 °C for 24 h. While *Staphylococcus aureus* ATCC 29213, *Pseudomonas aeruginosa* ATCC 27853, *Streptococcus faecalis* ATCC 8043*, Bacillus cereus* ATCC 33018*, Escherichia coli* ATCC 25922, *Listeria monocytogenes* ATCC 7644, *Salmonella serovar* Paratyphi B. ATCC 10719 and *Proteus mirabilis* ATCC 7002, were provided by central laboratories in Cairo and used as indicator strains. Indicator strains were cultivated in nutrient agar medium at 37 °C for 24 h.

### Confirmation of the antibacterial activity

For the preparation of crude bacteriocin, 10 mL of MRS broth was inoculated with 0.1 mL of a freshly prepared culture of isolated strains and were incubated for 18 h at 37 °C. The grown culture was centrifuged at 4000×*g* for 4 min at room temperature in the centrifuge tubes. Five milliliters of the supernatant was mixed with catalase (Sigma) (5 mg/mL) on pH 7.0 to eliminate the effect of hydrogen peroxide produced by the strain and incubated at 37 °C for 2 h and the pH was adjusted to 6.5 using 1 M NaOH, then the supernatant was heated at 80 °C for 10 and filter sterilized, to obtain the crude bacteriocin [29, 30]. To assess the protein nature of antimicrobial substance, 5 mL of supernatant was mixed with proteinase K (Sigma) at (5 mg/mL) and incubated at 37 °C for 2 h. The enzyme reaction was terminated by boiling water bath for 3 min [26]. This treated supernatant was tested with crude bacteriocin by spot diffusion method [31]. The concentration of *S. aureus* as indicator strain was matched against 0.5 Mcfarland Standard in 100 mL peptone water and then diluted again (1:100) to obtain a concentration of 1.0 × 10^6^ CFU/mL. A lawn of the indicator strain was made by spreading the cell suspension over the surface of Mueller Hinton agar plates with a sterile cotton swab. 10 μL of crude bacteriocin, enzyme-treated supernatant, positive (gentamicin 10 μg) and negative (sterilized MRS broth) controls were spotted on the surface of inoculated agar, and plates were incubated overnight at 37 °C for 24 h. After incubation, inhibition was indicated by a clear zone around spots. This test was performed in triplicate.

## Detection and sequencing of the bacteriocin gene (*pln*EF)

### Detection of the bacteriocin gene

Detection of the *plnEF* gene was carried out previously [28], according to the method of Rizzello et al. [27], but the method is mentioned briefly for confidence. The strains were sub-cultured on MRS medium and incubated at 30 °C for 48–72 h. After extracting and purify genomic DNA from LAB strains (QIAamp DNA mini kit, Dalian, China). Species-specific *recA* primer was used to detect the *pln*EF gene [27] is shown in Table 1. PCR conditions as follows: 25 μL of reaction mixture containing 6 μL of bacterial DNA template, 1 μL of each primer and 12.5 μL of Emerald Amp GT PCR mastermix (2× premix), 4.5 μL of PCR grade water. While the thermal cycling program comprised of an initial denaturation step at 95 °C for 20 s and 40 cycles of 95 °C for 20 s, 60 °C for 40 s and 72 °C for 50 s. Melting curve analysis was performed at 95 °C for 15 s, 75 °C for 1 min and 95 °C for 15 s to assess the specificities of the amplifications. The electrophoresis was used to visualize the amplified products using 1.5% agarose gels stained with 0.5 μg/mL ethidium bromide. The obtained bands were photographed by a gel documentation system [32], and the data were properly interpreted through computer software (reference lab for veterinary quality control on poultry production, Animal Health Research Institute, Egypt).

### Sequencing of the bacteriocin gene

The band corresponding to the correct size of the bacteriocin gene was purified from the gel using QIAquick® PCR Purification kit (Qiagen, Germany) following the manufacturer instructions. A purified RT-PCR product was sequenced in the forward and/or reverse directions on an Applied Biosystems 3130 automated DNA Sequencer (ABI, 3130, USA). A BLAST® analysis (Basic Local Alignment Search Tool) (http://www.ncbi.nlm.nih.gov/BLAST) was initially performed to establish sequence identity to GenBank accessions. The sequence reaction was done according to the instruction of the manufacture.

#### Phylogenetic analysis

Phylogenetic analysis of sequences was carried out using the Clustal W multiple sequence alignment program (version 1.83 of MegAlign module of Lasergene DNAStar software Pairwise) and Phylogenetic analyses were done using a neighbor-joining tree with maximum composite likelihood substitution model in MEGA6. Numbers at nodes represent measures of robustness depend on 1000 bootstrap replications [33].

#### Physico-chemical properties and 3D structure analysis of plnEF

Some of the physicochemical properties like isoelectric point, molecular weight, net charge at pH 7, and grand average of hydropathicity (GRAVY) of the identified peptides plantaricin E and F were estimated directly from the ProtParam website (http://web.expasy.org/protparam/) [34]. Whereas, 3D structure prediction and protein homology analysis were carried out by using the Phyre2 web portal for protein modeling (http://www.sbg.bio.ic.ac.uk/servers/phyre2/html/) [35]. In addition to the prediction of the alpha helix content of the pln EF peptide by using Hierarchical Neural Network (HNN) website( https://npsa-prabi.ibcp.fr/cgi-bin/secpred_hnn/) [36].

### Extraction of the bacteriocin (EX-PL)

The bacteriocin was precipitated from 80 mL crude extract by the addition of Ammonium sulfate in a cold condition (temperatures of 5 °C to 10 °C), while stirring gently to achieve 80% saturation and was then left overnight. The precipitate was then separated from the filtrate by centrifugation (Hitachi–CS150FNX) at 8.000 rpm for 10 min. Then, the precipitate was dissolved in 2 mL distilled water, the finally obtained protein was 40× concentrated [37]. Total protein concentration was detected using a colorimetric method [38], and the absorbance was measured within 30 min at 546 nm wavelength.

### Synthesis of plantaricin-incorporated silver nanoparticles (PL-SNPs)

Synthesis of silver nanoparticles (SNPs) was carried out by the method described by Adebayo-Tayo et al. [39] with slight modifications. PL-SNPs were prepared at room temperature by successive addition of freshly prepared 3 mM silver nitrate (15 μL) and 0.6 mM NaBH4 (100 μL) to 1 mL extracted plantaricin solution in water. Also, a free AgNB solution (without bacteriocin) was prepared. The obtained mixture was exposed to natural visible light. After 30 min, the degree of the mixture color turned from colorless to brown, which reflected the synthesis of silver nanoparticles.

### Scanning electron microscopic analysis (SEM) and energy dispersive X-ray (EDX)

The characterization of the synthesized bacteriocin incorporated silver nanoparticle was examined by SEM (model QAUNTA FEG250) to define the morphology of the nanoparticles. For the preparation of the tested sample, a drop of nanoparticle solution was added over the carbon tape glued over metal stub and was then covered with gold and examined at × 10,000 magnification then analyzed by EDX at 20 kV to accurately define the elemental constitution of the formed particles. AZTEC software (Oxford Instruments NanoAnalysis, Ver 1.2) was used for the analysis, while ImageJ software (Version 1.38) was used for particle size determination [40].

### Evaluation of the antibacterial activity of the EX-PL and PL-SNPs

#### Spot diffusion method

The antibacterial activity of the EX-PL and PL-SNPs produced from *Lb. plantarum* strain were screened against eight indicator strains by the spot diffusion method as described previously.

#### Determination of the antibacterial activity titer

The antibacterial activity titer was determined according to the well diffusion method [41]. *S. aureus* was used as an indicator strain. The MHA plates seeded with 100 μL suspension of previously adjusted indicator strain (10^6^) were allowed to dry and a sterile cork borer of diameter 5 mm was used to cut uniform wells in the agar. Two-fold serial dilutions of free AgNB solution, an extracted (EX-PL) and a plantaricin-incorporated silver nanoparticles were prepared. Each well was filled with 50 μL of each dilution. Eventually, after the incubation period (37 °C for 24 h), the zone of inhibition was measured. The antimicrobial activity of bacteriocin was defined as the reciprocal of the highest dilution showed the inhibitory activity and was expressed as arbitrary units per milliliter (AU/mL), calculated according to following formula: a^b^ × 20, where “a” is equal to 2, and “b” the number of the wells containing the dilution that produced the inhibition zone.

#### Minimum inhibitory concentration (MIC)

The minimum inhibitory concentration (MIC) was determined by the broth macro-dilution method [42]. In this method, different suitable stock concentrations were made from each solution including extracted bacteriocin, bacteriocin-incorporated silver nanoparticles, and free silver nanoparticles (AgNPs). Then, a series of two fold dilution was prepared in 1 mL MH broth. By transferring 1 mL of each preparation to 1 mL broth. This was repeated to complete the serial dilution. Then, each dilution was inoculated with 100 μL of the indicator strain (final concentration in each tube was approximately 5 × 10^5^ CFU/mL followed by incubation for 24 h at 37 °C. A positive control tube containing only 1 mL of two fold diluted media plus 100 μL of the indicator strain and a negative control containing only media were also prepared. MIC was the final dilution of tested bacteriocin that able to inhibit the visible growth of the indicator strain and was expressed also as mg/mL [43].

#### Minimum bactericidal concentration (MBC)

MBC was determined from those dilutions that showed no bacterial growth in the MIC test. 10 μL was spotted on a MHA plate and incubated (37 °C for 24 h). The lowest concentration showing no growth of bacteria was considered as MBC.

### Stability of the antibacterial activity

For testing the stability of extracted and bacteriocin-incorporated silver nanoparticles during storage at 4 °C, 10 μL was taken from the stored material and the bacteriocin activity was determined using agar well diffusion test weekly for 2 months [41].

### Statistical analysis

Antibacterial activity tests were performed in triplicate and the results were presented as mean values ± standard deviation. The statistical relevance was assessed by Student’s *t* test. The statistical significance level was defined as *P* > 0.05.

## Results

### Confirmation of the identity of *Lb. plantarum*

**Fig. 1:**
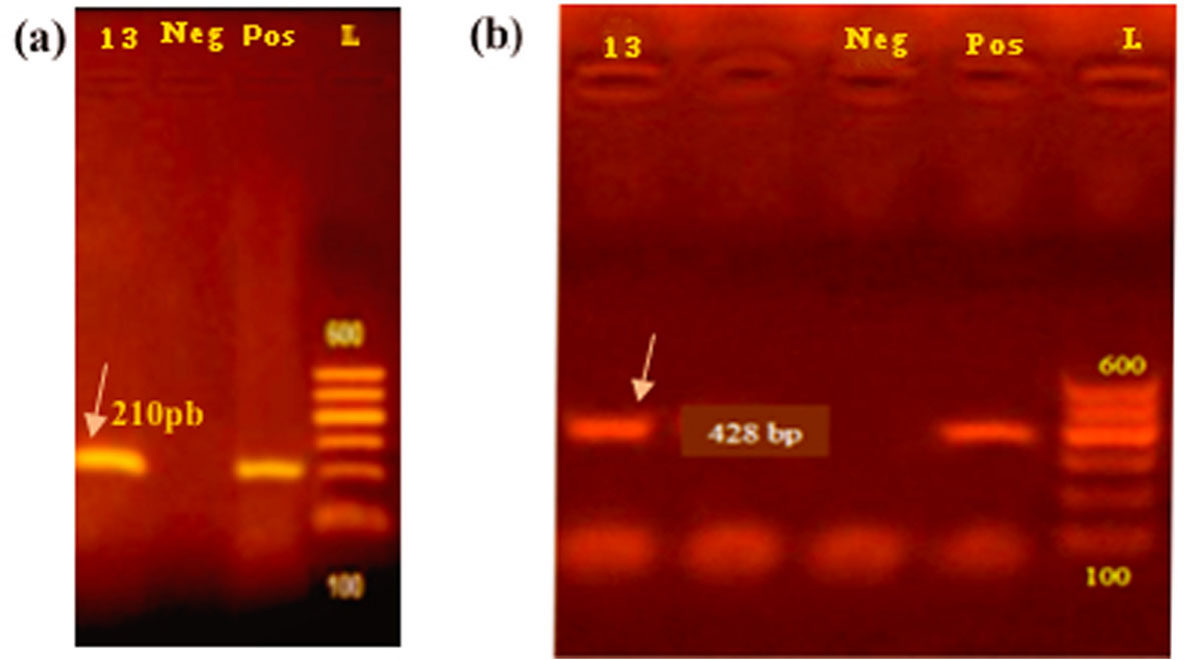
Amplification products obtained from Lactic acid bacterial isolates using the *rec*A gene primers. **a** The lane product of the gene encoding *Lb. plantarum* indicated by the arrow. **b** The lane product of *pln*EF gene from the strain-no. 13 indicated by the arrow, using primers pairs; lane L: 1000 bp ladder. Lane Neg: negative control, Lane Pos: positive control

Lactic acid bacterial isolate no. 13 was confirmed to be *Lb. plantarum* by PCR specific primers and gives an amplification product of 210 pb (Fig. 1a).

### Molecular characterization of *pln*EF bacteriocin gene

The PCR results showed that the fragment with 428 bp of *pln*EF gene of *Lb. plantarum* strain was successfully obtained (Fig. 1b). *Lb. plantarum* 13*plnEF* Egypt 2018 was deposited in NCBI under the GenBank accession number of MN172264.1.

**Fig. 2:**
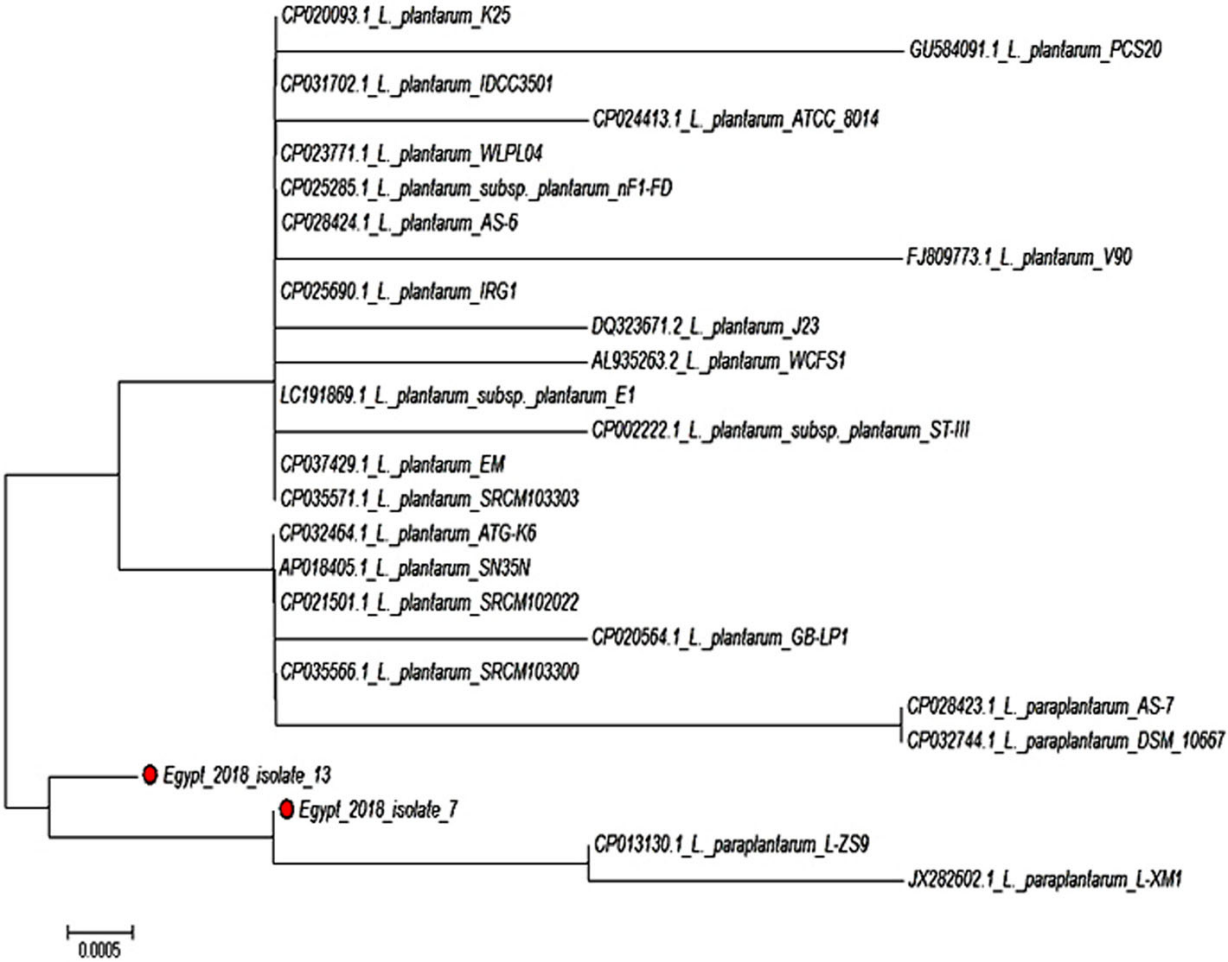
Phylogenetic tree based upon the neighbor-joining of DNA gene sequences of *Pln*EF gene for *Lb. plantarum* strain isolate no. 13

**Fig. 3:**
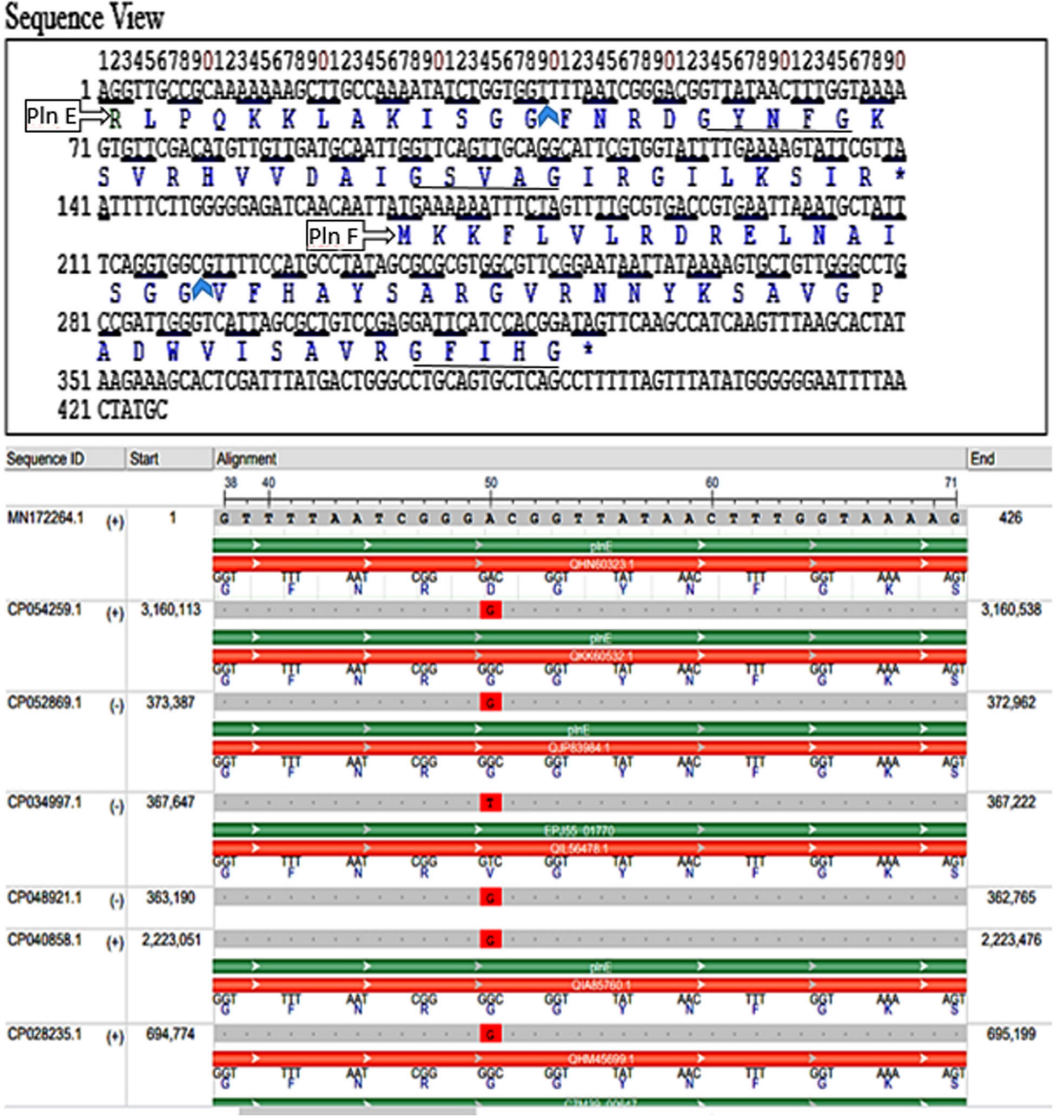
The DNA sequence of the gene encoding plantaricin EF of *Lactobacillus plantarum* strain EG.LP.18.13 (MN172264.1). **a** Nucleotide and deduced amino acid sequences of *pln* EF; blue arrows indicate the cleavage sites in the PlnE and PlnF pre-mature peptides and the starts of the corresponding mature peptides, stop codons are indicated by asterisks at the ends of the protein sequences; GXXXG motives are underlined. **b** Multiple sequence alignment view of nucleotides of pre-mature fragment of plnE with other closely plnE peptides producing *Lactobacillus* strain references showing the dissimilar amino acids in red

The similarity percent of the nucleotide sequence of the *pln*EF Egypt18 loci obtained from *Lb. plantarum* recorded 99.77% homology with *pln*EF locus of other *Lb. plantarum* references strain aligned such as *Lb. plantarum* strain AMT74419, LS/07, and X7022 (Table 2). Figure 2 represents the phylogenetic tree based upon the Neighbor-Joining of DNA gene sequences of *pln*EF gene for *Lb. plantarum* strain isolate no. 13. As can be seen from Fig. 3a, the *pln*EF composed of two genes *pln* E and *pln* F that encoding two peptides and are located next to each other. Based on the gene components obtained from the BLAST® analysis that composed loci of *pln*EF, *pln*E gene was only differed by one nucleotide at position 50 (adenine > guanine) from other closely related strains except for *Lb. plantarum* strain LS/07 (accession: CP034997.1); the replacement was in (adenine > thymine) as indicated in Fig. 3b.

**Table 2:** Percent of the similarity of *pln*EF gene (*Lb. plantarum* strain EG.LP.18.13)

Species	Percent of similarity	gi| Accession number
*Lb. plantarum* strain TCI507	99.77%	gi|1851159953|CP054259.1
*Lb. plantarum* strain AMT74419	99.77%	gi|1837877912|CP052869.1
*Lb. plantarum* strain LS/07	99.77%	gi|1821205466|CP034997.1
*Lb. plantarum* strain X7022	99.77%	gi|1812602472|CP048921.1
*Lb. plantarum* strain 202195	99.77%	gi|1809139426|CP040858.1
*Lb. plantarum* strain SRCM101511	99.77%	gi|1802495744|CP028235.1
*Lb. plantarum* strain SRCM102737	99.77%	gi|1799535273|CP028261.1
*Lb. plantarum* strain SRCM101105	99.77%	gi|1799509686|CP028222.1
*Lb. plantarum* strain SRCM100995	99.77%	gi|1799506451|CP028275.1
*Lb. plantarum* strain 8P-A3	99.77%	gi|1785299055|CP046726.1
*Lb. plantarum* strain 123-17	99.77%	gi|1784839328|CP046656.1
*Lb. plantarum* strain TMW	99.77%	gi|1773397096|CP021929.1
*Lb. plantarum* strain pc-26	99.77%	gi|1699908677|CP023301.1
*Lb. plantarum* strain J26	99.77%	gi|1611638273|CP033616.1
*Lb. plantarum* strain EM	99.77%	gi|1588044627|CP037429.1
*Lb. plantarum* strain EM	99.77%	gi|1584026770|CP025690.1
*Lb. plantarum* strain EM	99.77%	gi|1584026770|CP025690.1
*Lb. plantarum* strain TCI507	99.77%	gi|1851159953|CP054259.1

*gi* GenInfo Identifier

The gene plnE encodes a propeptide with 46 aa protein, Accession: QHN60323.1, with sequence of “**RLPQKKLAKISGGFNRDGYNFGKSVRHVVDAIGSVAGIRGILKSIR**.” While the other subunit is the gene plnF that encodes a propeptide (QHN60324.1) with 52 residue with a sequence of “MKKFLVLRDRELNAISGG**VFHAYSARGVRNNYKSAVGPADWVISAVRGFIHG**.”

Also, it was demonstrated that the mature pln E peptide contains two moieties of GXXXG at positions 5 and 20. Whereas pln F contains one moieties of GXXXG at position 30 (Fig. 3a).

While the BLASTX protein database homology search on the deduced mature peptides of pln EF nucleotides showed 100% homology to plantaricin EF for subunit PlnF and PlnE with accession no. WP 003643811.1 and WP 033611266.1 among other closely related references, respectively.

**Fig. 4:**
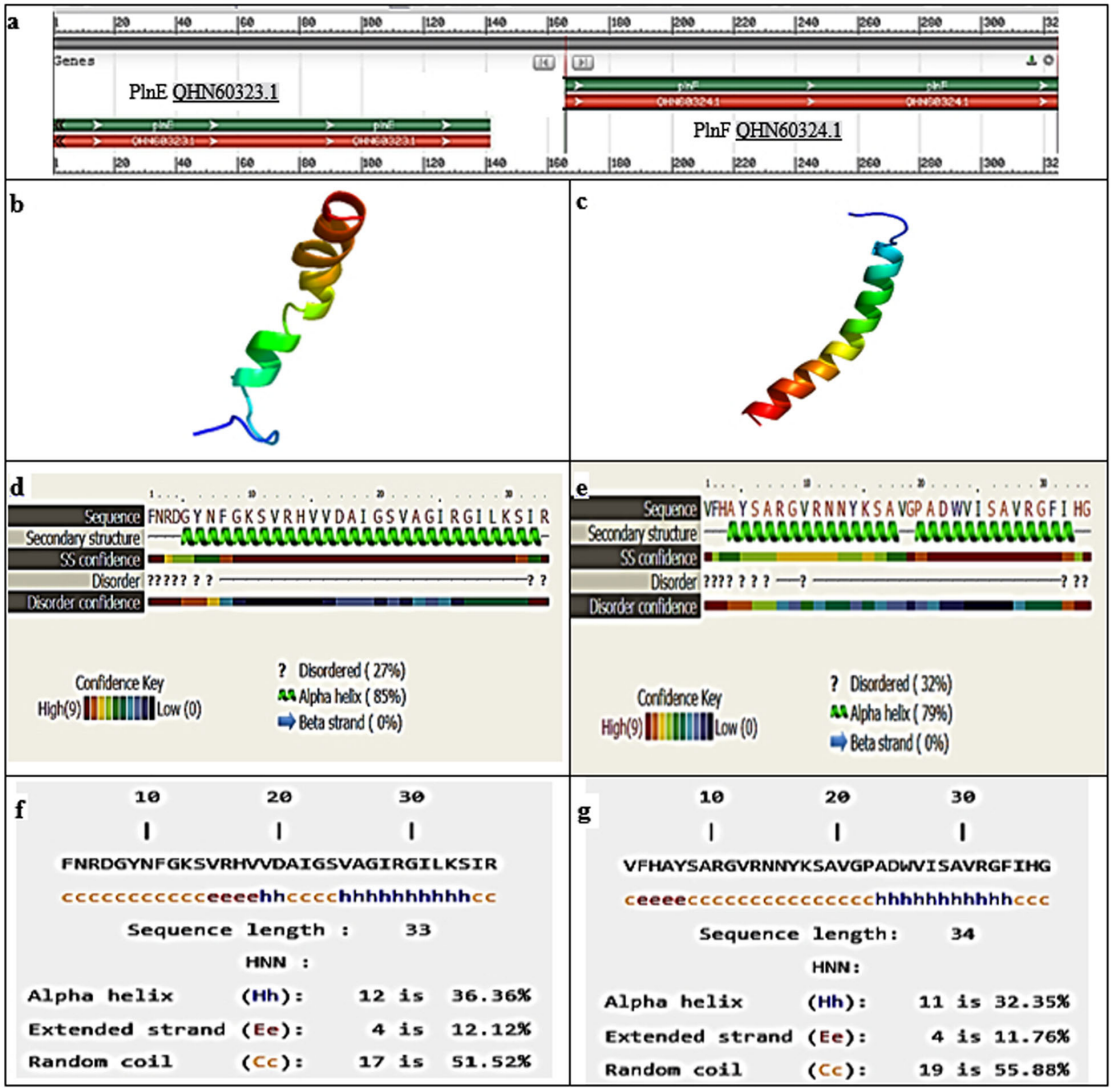
EG.LP.18.13 *pln* EF gene sequence and bioinformatics on its mature peptides. **a** A graphic of pln EF encoding genes (MN172264.1) represent plnF and plnE. **b**, **c** 3D-structure predicted by the Phyre 2: image colored by rainbow N (blue color)→C terminus (red color); green helices represent α-helices, blue arrows indicate β-strands and faint lines indicate coil (**d**) and (**e**) are HNN secondary structure confidence analysis of plnE and plnF peptides, respectively: the ‘SS confidence’ line indicates the confidence in the prediction; red : high confidence; blue: low confidence. **f**, **g** Prediction of α-helix contained within each peptide of plnE and plnF, respectively. **h** α-helix; **c** random coil; **e** extended strand

The physico-chemical properties and structure of the deduced mature peptides carried out through computational tools revealed that plnE has 33 amino acids residue with theoretical molecular weight of 3.6 kDa, isoelectric point of 11.2 with a slightly negative GRAVY score (− 0.027). While plnF mature peptides has 34 residue amino acids residue with theoretical molecular weight of 3.7 kDa, isoelectric point of 10.6 with a positive GRAVY score. The 3D structure predicted with Phyre 2 (Fig. 4b, c) has been modeled with 99.8 and 99.9% confidence by the single highest scoring template (c2juiA) and (c2rlwA) for plnE and plnF peptides, respectively. The secondary structure predicted by the Phyre 2 gave one α-helical-like regions (residues 10–31) for pln E with alpha helical content of 85%, whereas it gave one long helix from residue 7 to 32, with alpha helical content of 79% for pln F (Fig. 4d, e). Meanwhile, the prediction of HNN gave α-helical content of 36 and 32.35% for pln E and pln F (Fig. 4f, g), respectively.

### Antibacterial activity of crude bacteriocin and total protein concentration

The antimicrobial activities of *Lb. plantarum* strain were checked by the spot diffusion method against *S. aureus*. It gives an inhibition zone of 11.5 mm after the elimination of other factors like acidity and hydrogen peroxide. The inhibitory activity of crude preparations was completely lost after the treatment with proteinase K that confirmed the protein nature of the inhibitory substance. Bacteriocin from *Lb. plantarum* was successfully extracted by the ammonium sulfate method. The total protein content was 60.9 mg/mL according to the colorimetric method endpoint assay.

### Characterization of plantaricin-incorporated silver nanoparticles (PL-SNPs)

#### Color change

PL-SNPs formation was confirmed by the color change; the appearance of brown color in the solution containing the biomass confirmed the formation of silver nanoparticles.

#### SEM

**Fig. 5:**
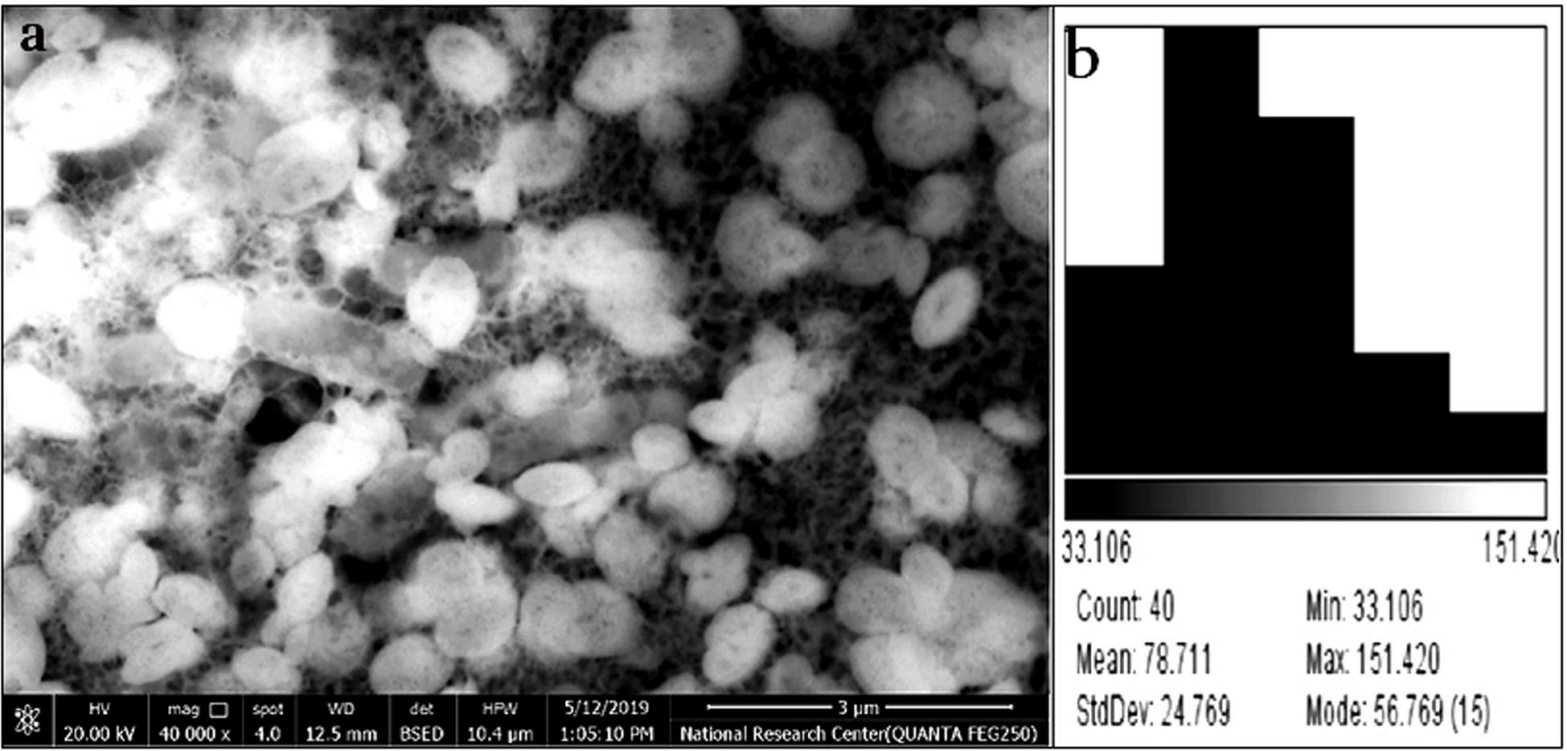
Plantaricin-incorporated silver nanoparticles PL-SNPs. **a** The SEM micrographs of PL-SNPs. **b** Histogram data of particles dimensions (nm) PL-SNPs using ImageJ

The SEM micrographs of Plantaricin-incorporated Silver nanoparticles of PL-SNPs showed high density and spherical shaped nanoparticles with a mean diameter of 78.7 nm (Fig. 5).

#### Analysis through energy dispersive X-ray (EDAX) spectrometers

**Fig. 6:**
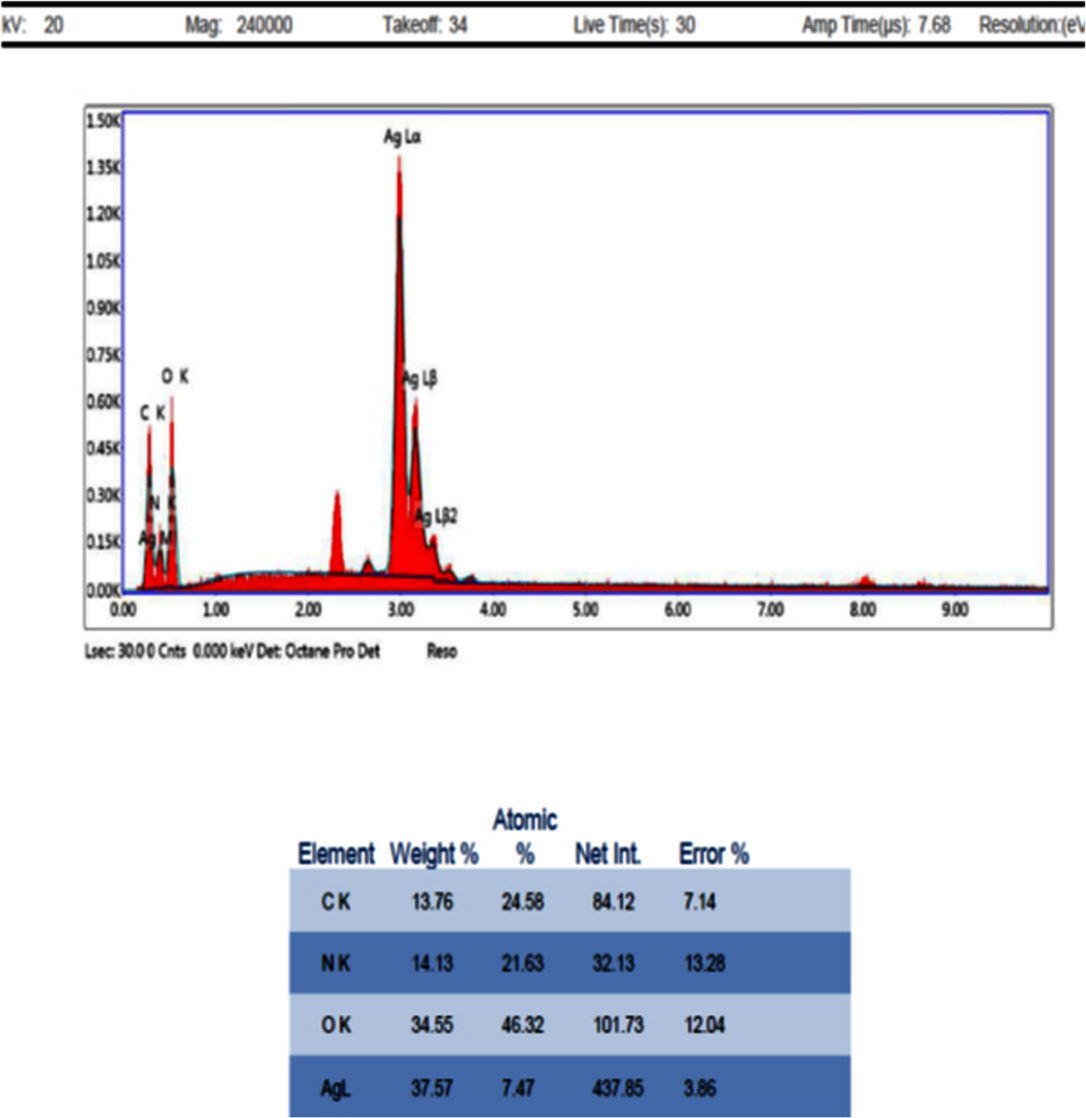
EDAX micrograph of plantaricin-incorporated silver nanoparticles (PL-SNPs), C: carbon content, N: nitrogen content, O: oxygen content, Ag: silver content

The EDAX graph of the PL-SNPs13 shows strong peak of silver element and smaller peaks for C and N content, confirmed the presence and the identification of elemental silver signal (Ag ɑ) (Fig. 6), thus giving confidence that silver has been correctly identified.

### Antibacterial activity of EX-PL and PL-SNPs

The susceptibilities of Gram-positive and Gram-negative bacteria to the growth inhibition by both EX-PL and PL-SNPs are presented in Table 3. EX-PL displayed inhibitory ability against seven indicators including *E. coli*, *S. aureus*, *Ps. aeruginosa*, *Salm.* Paratyphi *B*, *Strep. faecalis*, *B. cereus*, and *L. monocytogenes*. Among these the maximum activity was observed against *E. coli* when compared with other indicator strains. Extracted bacteriocin EX-PL did not have inhibitory activity against *L. monocytogenes*. However, the PL-SNPs show more inhibition towards all tested indicators including *L. monocytogenes* except for *P*. *mirabilis* which recorded the lesser inhibition zone (Table 3).

**Table 3:** The antibacterial activity of extracted plantaricin (EX-PL) and plantaricin-incorporated silver nanoparticles (PL-SNPs) expressed as diameter of inhibition zones (mm)

Indicator strains	Extracted plantaricin	Plantaricin-incorporated silver nanoparticles
***L. monocytogenes*** ATCC 7644	0.0 ± 0.0	14.5^a^ ± 0.0
***P. mirabilis*** ATCC 7002	10.5^a^ ± 0.4	6.5 ± 0.3^c^
***E. coli*** ATCC 25922	15.0^c^ ± 0.3	19.0^a^ ± 3.0
***S. aureus*** ATCC 29213	11.5^c^ ± 0.0	15.6^a^ ± 0.3
***Ps. aeruginosa*** ATCC 27853	11.5^c^ ± 0.1	14.0^a^ ± 0.1
***Salm. Paratyphi B*** ATCC 10719	12.6^b^ ± 0.4	14.5^a^ ± 0.3
***Strep. faecalis*** ATCC 8043	12.0^c^ ± 0.2	15.0^a^ ± 0.3
***B. cereus*** ATCC 33018	8.5^d^ ± 0.3	14.5^a^ ± 0.5

Different letters (a, b, c) in the same raw are significantly different (*p* < 0.5)

### Antibacterial activity titers, minimum inhibitory concentration (MIC), minimum bactericidal concentration (MBC) and stability

**Fig. 7:**
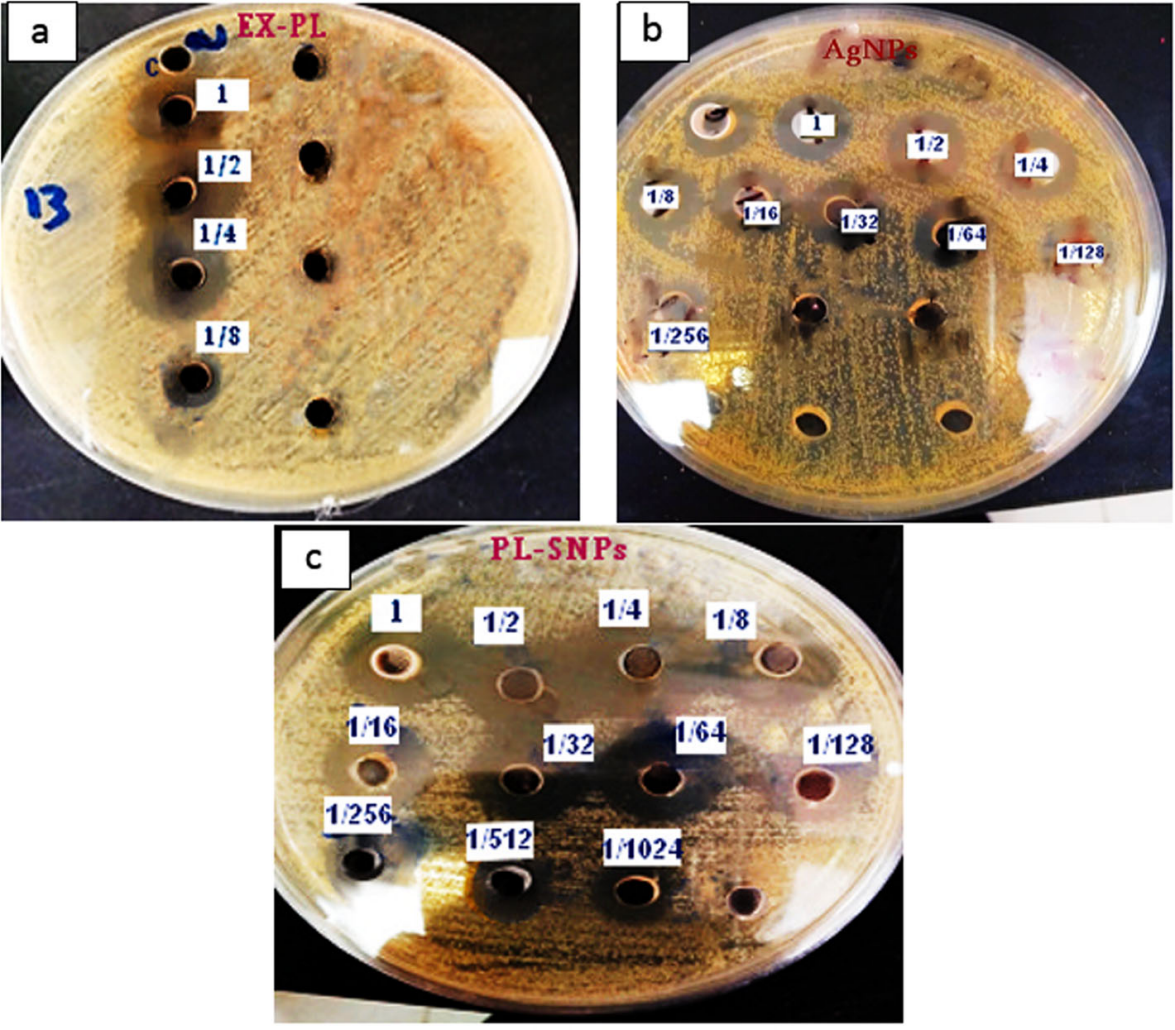
The arbitrary unit detection of bacteriocin using well diffusion method, against *S. aureus*. **a** Extracted bacteriocin EX-PL. **b** Free AgNPs solution. **c** Plantaricin-incorporated silver nanoparticles PL-SNPs

The results for Bacteriocin titers (AU/mL) and MIC against *S. aureus* as an indicator strain are presented in Fig. 7 and Table 4, respectively. The antibacterial activity was remarkably increased by the application of nanotechnology; as it was 160 AU/mL for EX-PL, 5120 AU/mL for free AgNPs and increased up to 20480 AU/mL for PL-SNPs. Likewise, using nanotechnology has considerably decreased the MIC of EX-PL against *S. aureus* as an indicator strain. The MIC of EX-PL was 7.6 mg/mL, while it was 0.004 mg/mL for PL-SNPs. Whereas MBC was 15.22 mg/mL for EX-PL which was decreased to 0.625 mg/mL for PL-SNPs. While MIC and MBC for AgNPs were 0.39 and 12.5 μg/mL, respectively. The stability of plantaricin-incorporated silver nanoparticles and natural plantaricin was measured periodically. The stability at 4 °C for EX-PL was 5 days; however, it was increased to 60 days for PL-SNPs.

**Table 4:** MIC, MBC and the stability during 60 days of natural bacteriocin and nano-silver bacteriocin against *S. aureus*

Tested types	Total antibacterial activity (AU/mL	Specific activity (AU/mg)	MIC	MBC	Stability (day)
			mg/mL	mg/mL	Stored at 4 °C
EX-PL	160	2.62	7.6	15.22	5
PL-NSPs	20480	4096	0.0048	0.072	60
AgNPs	5120	-	0.0195	0.3125	15

Specific activity is the activity units divided by the protein concentration (mg/mL)

## Discussion

*Lb. plantarum* is the most valuable species with beneficial properties, which is abundant in various habitats such as milk and cheese. *Lb. plantarum* strains have been previously isolated from dairy products of Luxor-Egypt [28]. Strain no. 13 has been chosen for its potential antibacterial activity. The antibacterial activity was proved to be a result of bacteriocin production by the elimination of other antibacterial factors, including acidity and H_2_O_2_. Bacteriocins synthesis by LAB may be an adept form particularly towards the environmental conditions and microbial interactions [44, 45].

In the attained study, *Lb. plantarum* was confirmed to have *pln*EF gene, which is responsible for the production of plantaricin (pln) EF [28]. PlnEF is the most well-characterized in class IIb bacteriocins [46]. To understand the nature of bacteriocins as antimicrobial agents requires detailed about how bacteriocins function, so it is necessary to investigate their 3D structures, as bacteriocins function through structural interactions. It was pointed out that the genes encoding the two peptides of class IIb bacteriocins are located next to each other on the same operon and formed in equivalent quantities, which explains the fact, that the two-peptide bacteriocins interact in a functional structural inducing manner upon exposure to the membrane of the target cells [47]. These pre-peptides with double-glycine leader sequences are cleaved off during export to produce active mature peptides with sizes of 33 aa (PlnE) and 34 aa (PlnF). In the present study ,the 3D structures of the sequences of the two peptides of plantaricin EF as predicted by Phyre 2 homology modeling displayed sequence similarity with the structures of PlnEF elucidated by Fimland et al. [48]. The confidence score of Phyre2 denoted the likelihood of homology and a score > 90%; indicated that the query protein adopts the overall same fold as the template [35]. From former structural studies, it have been established that the bacteriocin peptides are all unstructured in aqueous solutions, but both plnE and plnF can form amphiphilic α-helices when get in contact with a membrane. Gravy score is used to represent the hydrophobicity value of a peptide and is calculated from the sum of the hydropathy values of all the amino acids divided by the sequence length [34]. In the present results pln E gave a negative value which means hydrophilic, while pln F gave a positive value which indicates hydrophobic. These results is in a good agreement with Fimland et al. [48] who pointed out that the two helices of plnE are both amphiphilic, while only the C-terminal part of the plnF helix is amphiphilic and the N-terminal part is polar. Additionally, Ekblad et al. [49] have predicted that the two peptides PlnE and PlnF act together in an antiparallel manner and the C-terminus of PlnE and N-terminus of PlnF are located on the external part of target cell membranes and the N-terminus of PlnE and C-terminus of PlnF stand on the inner part.

The amphiphilic α-helices detected in plnEF are important motifs in most of two-peptide bacteriocins [48, 49]. Helicity was evidenced to play an essential role in the specificity and activity of the peptides. Determination of α-helices in Phyre 2 is not completely accurate as it considers both π-helices and 3_10_-helices as α-helices. Accordingly, we used another computational program (HNN) which discriminated the types of helix in the secondary structure presenting pln E with a 36 and pln F with 32.35% α-helices content, respectively. It is noteworthy to till that the previous CD-structural studies yielded a helical content of about 39% and 67% in plnE and plnF, respectively [48].

In the current study, the detection of GxxxG motifs in the sequence of *pln*EF is commonly recognized in the class IIb bacteriocins sequences. GxxxG motifs have shown to be essential for conjugating and facilitated helix-helix interfaces and enabled penetration of the target cell membranes [50]. PlnE has two GxxxG motifs, one at residues 5 to 9 and one at residues 20 to 24. While plnF has one such motif at residues 30 to 34. The peptides are flexible in these GxxxG regions. Ekblad et al. [49] illustrated that the replacement of glycine residues in the GxxxG motifs might result in the loss of the peptide activity. So, GxxxG might affect the peptide’s biological activity. It is worth to mention that the substitution in the nucleotides in the present study led to translation of aspartic acid (Asp-D) instead of glycine (Gly-G) at the N-terminal region of pln E. Aspartic acids is acidic, polar and negatively charged, that facilitates interaction with positively charged metals ions. In addition that it contains a shorter side chain making it more rigid to some extent within protein structures and more likely to be involved in protein active sites. However, glycine is uncharged and contains only a hydrogen atom as a side chain. That glycine gives a flexibility within protein structure. This replacement cause a decrease in the positive net charge, thus may be suggested to have an effect on decreasing antibacterial activity. However, the substitution of Gly-G to Asp-D is unlikely to cause an alteration in the physicochemical properties of peptides, because these amino acids are mostly prefer to turn regions on the surface of the protein; aspartate favors to expose their charged side chains to solvent [51]. Also as can be noticed, this replacement is neither fall in GXXXG motifs nor in helix regions so it is not assumed to affect the function of peptides.

Because of its high solubility and yield, Duong-Ly and Gabelli [52] specified, ammonium sulfate as the most proper salt for initial purification step for large volumes of proteins. Song et al. [53] and Chen et al. [54] used ammonium sulfate precipitation method for purification of the plantaricin from *Lb. plantarum* ZJ5 and plantaricin ZJ316, respectively. In the current results, the extracted protein possessed antibacterial activity of 160 AU/mL, these results exhibited similarity to what obtained for extracted plantaricin LPL-1 with a total activity of 204 AU/mL [55] and extracted plantaricin MG with a protein concentration of 59 mg/mL and an activity of 320 AU/mL [56]. In a more recent study, plantaricin GZ1-27 was extracted by the same method resulting in a protein concentration of 102.5 mg/mL with a total activity of 2.69× 10^4^ IU/mL [57].

It was pinpointed that there are limiting factors that hurdle the activity of natural bacteriocin, including the rapid biodegradation within 3 days in the environment, high effective concentration accompanied with a low yield [58]. Nanotechnology is considered one of the modern methods that have proven to overcome most of these defects. Lazzari et al. [59] demonstrated that nanoparticles possess good stability in biological fluids, besides their effective antimicrobial activities results from the high surface area, so incorporation of nanotechnology in bacteriocin encapsulation to improve its characteristics, could be considered the best choice [60]. Accordingly, we have applied nanotechnology for the production of bacteriocin-incorporated silver nanoparticles using the extracted plantaricin and compared the antibacterial activity of extracted plantaricin with its nanoparticle formulations. PL-SNPs was successfully synthesized, and that was confirmed by changing in color as a result of chemical reduction of silver nitrate with sodium borohydride and the formation of Ag+ which give brown color upon exposure to sunlight [19]. EDAX is an analytical technique used for the elemental analysis or the chemical characterization of a sample and it relies on the interaction of some source of X-ray excitation. EDAX spectral analysis of P-SNPs preparations gave a strong silver signal, typical for metallic silver, also weaker signals related to carbon, oxygen, and nitrogen were recognized which might originate from bacteriocin incorporated structures bound to the AgNPs surface. It was elucidated that hydroxyl and amino groups which comprises the active functional groups of bacteriocin, can be chelated by silver, and by this means it could cover a substantial portion of the surface of AgNPs [23]. With regard to SEM, it was revealed that the PL-SNPs particles have approximately spherical shapes with approximately mean diameter of 78.7 nm, which was approximately bigger than the mean diameter of 35 nm reported for the enterocin-coated silver nanoparticle [61].

It is already accomplished that the bacteriocin produced from *Lb. plantarum* demonstrated a potent antimicrobial activity [62, 63]. Although EX-PL in the current study displayed antibacterial activity against the seven studied indicators, it did not display activity against *L. monocytogenes*. Interestingly, nanomaterial’s encapsulation boosted the antibacterial activity of extracted bacteriocin against the majority of tested indicator strains including *L. monocytogenes*. These findings are in accordance with Arakha et al. [64] who have found that interfacial assembly of nisin at silver nanoparticles enhanced its antibacterial efficacy against nisin-resistant organisms. Furthermore, the total antibacterial activity of the extracted bacteriocin was increased by 99.6% with a great reduction of MIC values (4 μg/mL) against *S. aureus* after incorporation into silver nanoparticles as compared to the free bacteriocin or AgNPs. In the current results, the antibacterial activity of free AgNPs was higher than extracted plantaricin and lower than synthesized PL-SNPs, which strongly implied that nano-formulation showed enhanced antibacterial efficacy*.* Pal et al. [65] revealed that the conjugated antibacterial peptides with nanoparticles exhibited improved potency by 80% more against all the tested strains of *Ps. aeruginosa* and *Klebsiella pneumonia*. However in another study, the silver-encapsulated nisin recorded MIC of 60 μg/mL that gave approximately 22% increased activity than the non-encapsulated nisin [66]. Unfortunately, there are scarce studies addressing the incorporation of bacteriocins produced by *Lb. plantarum* in nanoparticles, for instance, the incorporation of a bacteriocin produced by *Lb. plantarum* ATM11 in conjugates containing gold nanoparticle resulted in enhanced antibacterial activity [67], as well as the microencapsulation of bacteriocin from *Lb. plantarum* SC01 in Alginate-Gelatin Capsules produced highly active compounds [68]. Whereas the encapsulation of plantaricin 423 in nanofibers led to a decreased antibacterial activity [69].

On the other hand, in the present results, it was noticed that the antibacterial activity of P-SNPs towards *P. mirabilis* was very lower than that of free bacteriocin; these results was in accordance with Abbaszadegan et al. [70] as they found that *P. vulgaris* was the most resistant test bacteria to silver nanoparticles, and they concluded that even though it is known that silver nanoparticles is more active against Gram-negative bacteria, its potential likewise depends on the types of bacterial species. Hence, the reported decreased antibacterial activity of PL-SNPs against this species is related mostly to conjugation with silver nanoparticles.

Another important finding was that PL-SNPs displayed a better stability than AgNPs and EX-PL as the antibacterial activity did not alter significantly over a period of 60 days, while the extracted bacteriocins are only suitable for short-term storage. These results in harmony with Fahim et al. [71] who found that the avicin-nanocomposite demonstrated antibacterial activity lasting for at least 24 days.

Despite there is a great interest today towards peptides-coated nanoparticles, the mechanism underlying interaction of the peptides with nanoparticles surface is still not yet fully understood [72]. Insights into the electrostatic properties might facilitate speculating such interaction. Herein, pln EF peptide is a cationic peptide; this cationic character enables the interaction with negatively charged phospholipids found in bacterial membrane. Also, it is established that both peptides are unstructured in water and becomes structured and form an amphiphilic α-helical structure upon contact with a membrane-mimicking environment especially with anionic liposomes and micelles. In fact, the α-helical content is increased in the presence of negative ambiances, which in turn leads to improving in the peptides’ reactivity against bacteria by permits the peptides to partition into the bacterial membrane lipid bilayer [48]. On the other hand, Abbaszadegan et al. [70] have declared that the charge of AgNPs has a significant effect on the antibacterial activity, as it have been found that there was a less activity detected with the negatively charged AgNPs, which is likely resulted from repulsion between both bacterial cells and AgNPs; however, the toxicity of negatively charged AgNPs is related mostly to the released amount of Ag^+^. Also it have been suggested that amphiphilic residue like arginine and lysine plays a significant role in the adsorption of the peptides on nanostructured surfaces with forming a well-arranged, lined domain structure [73, 74]. Hence, it is possible to suppose that the electrostatic charge play an important role in the first binding between peptides and silver nanoparticles through the amphiphilic residue in pln EF, which cause the formation of a more staple AgNPs by exerting a polyvalent effect thus preventing aggregation of particles [22, 75]. In the same way, the secondary structure of peptides would be expected to attain a more α-helix content. Subsequently, the coated AgNPs can efficiently attach negatively charged bacterial cells through hydrophobic interactions, as it assumed that the hydrophobic side of the structure can pass in and disrupt the bacterial membrane [48]. Together with the fact that both pln EF and AgNPs initial killing steps are relaying on forming pores. Consequently, this will led to the synergistic antibacterial effect of plantaricin and silver nanoparticles through the increase in permeability of the bacterial cell membrane. With the presence of large numbers of adsorbed peptides that simplify the penetration of nanoparticles. Then, nanoparticles together with peptides induce several killing mechanism inside bacterial cells. This hypnosis accords with Sharma et al. [61] who anticipated that improvement of Enterocin-SNPs is due to the presence of enterocin molecules on the surface of SNPs that gave more chance for interaction with the bacterial surface. Besides it was recently interpreted that the enhanced antibacterial activity of the Bacteriocin/AgNPs bioconjugate is related to the increase in the disruption of cell membrane permeability, leakages of protein and DNA, and formation of interfacial and intracellular ROS production which exerted oxidative stress [64, 76].

## Conclusion

To the best of our knowledge, the existing study had succeeded in synthesizing plantaricin-incorporated silver nanoparticles which has not only enhanced antibacterial activity against tested foodborne pathogenic bacteria, but also exhibited good inhibitory activity against *L. monocytogenes*, that was resistant to the free plantaricin. Besides, extending the bacteriocin stability to about 2 months is considered a noteworthy addition. Hence, this nano-preparation could be efficiently applied in many practical approaches such as in the food industry or in the medical applications after wisely determining its safety for human use.

## Supplementary Information

**Additional file 1 **Detection of antibacterial activity and the protein nature of antimicrobial substance. **Figure 1s**. Antibacterial activity by disk diffusion methods showing: inhibition zones of CFS supernatant of *L. plantarum*, crude bacteriocin and absence of zone of protease treated-crude bacteriocin.

**Additional file 2 **NCBI Multiple DNA Sequence Alignment Viewer of *pln* EF encoding genes. Multiple alignment using BLAST of *Lactobacillus plantarum* strain EG.LP.18.13 plantaricin E (plnE) gene, partial cds; and plantaricin F (plnF) gene, complete cds GenBank: gi|1799633834|gb|MN172264.1|, Presenting the deuced amino acids dissimilarity in red color.

**Additional file 3 **NCBI Multiple protein Sequence Alignment Viewer of *pln* EF pre-mature peptides. Multiple alignment using BLAST plantaricin E (plnE) peptide (QHN60323.1) followed by plantaricin F (plnF) peptide (QHN60324.1), presenting amino acids dissimilarity in red color.

**Additional file 4.** Phyre Investigator output for plnE mature peptide with c2juiA template. Homology using Phyre2 of plnE peptide with template c2juiA and secondary structure prediction.

**Additional file 5.** Phyre Investigator output for PlnF mature peptide with c2rlwA template. Homology using Phyre2 of PlnF peptide with template c2rlwA and secondary structure prediction.

**Additional file 6.** Physico-chemical properties and 3D-structure of peptides. Ccomputational software used to analyses physico-chemical properties and 3D-structure of mature peptides pln E and pln F.

## Data Availability

Authors declare that all generated and analyzed data are included in the article. The genome sequence of Plantaricin *EF Egypt 2018 strain-No.13* has been deposited in GENBANK under Accession numbers of MN172264 [https://www.ncbi.nlm.nih.gov/nuccore/MN172264].

## Abbreviations

LAB: Lactic acid bacteria
GRAS: Generally recognized as safe
PCR: Polymerase chain reaction
rec A: Recombinase A
MRS: Man-Rogosa and Sharpe
CFU: Colony forming unit
pH: *Pondus hydrogenii* (quantity of hydrogen)
DNA: Deoxyribonucleic acid
*Lb*: *Lactobacillus*
NCBI: National Center for Biotechnology Information
SD: Standard deviation
SNPs: Silver nanoparticles
EN-SNPs: Enterocin-conjugated silver nanoparticles
PL-SNPs: Plantaricin-incorporated silver nanoparticles
