# Supplementary material for: Enhancement of the antibacterial potential of plantaricin by incorporation into silver nanoparticles
Source: J Genet Eng Biotechnol. 2021 Jan 20;19:13. doi: 10.1186/s43141-020-00093-z (PMC7817718; doi:10.1186/s43141-020-00093-z)
Supplement: Supplementary file 1 — Additional file 1 Detection of antibacterial activity and the protein nature of antimicrobial substance. Figure 1s. Antibacterial activity by disk diffusion methods showing: inhibition zones of CFS supernatant of L. plantarum, crude bacteriocin and absence of zone of protease treated-crude bacteriocin. [file 43141_2020_93_MOESM1_ESM.docx]

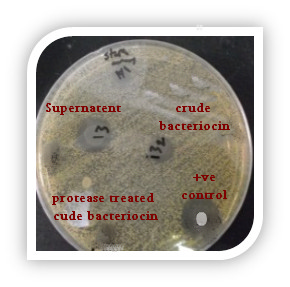


Figure 1s: - Detection of antibacterial activity and the protein nature of antimicrobial substance by disk diffusion methods: Presence of Inhibition zones for CFS supernatant of L. plantarum and crude bacteriocin. Absence of zone for protease treated-crude bacteriocin.
