# Supplementary material for: Enhancement of the antibacterial potential of plantaricin by incorporation into silver nanoparticles
Source: J Genet Eng Biotechnol. 2021 Jan 20;19:13. doi: 10.1186/s43141-020-00093-z (PMC7817718; doi:10.1186/s43141-020-00093-z)
Supplement: Supplementary file 3 — Additional file 3 NCBI Multiple protein Sequence Alignment Viewer of pln EF pre-mature peptides. Multiple alignment using BLAST plantaricin E (plnE) peptide (QHN60323.1) followed by plantaricin F (plnF) peptide (QHN60324.1), presenting amino acids dissimilarity in red color. [file 43141_2020_93_MOESM3_ESM.pdf]

NCBI Multiple Sequence Alignment Viewer, Version 1.18.0

Sequence ID		Start	Alignment (1–57)	End	Organism
QHN60323.1	(+)	1	R L P Q K K L A K I S G G F N R D G Y N F G K S V R H V V D A I G S V A G I R G I L K S I R	46	Lactiplantibacillus plantar...
WP_033611266.1	(+)	1	M L Q F E K L Q Y S R L P Q K K L A K I S G G F N R D G Y N F G K S V R H V V D A I G S V A G I R G I L K S I R	56	Lactiplantibacillus
AGE46854.1	(+)	1	R L P Q K K L A K I S G G F N R D G Y N F G K S V R H V V D A I G S V A G I R G I L K S I R	46	Lactobacillus paraplantar...
WP_003641985.1	(+)	1	M L Q F E K L Q Y S R L P Q K K L A K I S G G F N R G G Y N F G K S V R H V V D A I G S V A G I R G I L K S I R	56	Lactobacillaceae
WP_063845882.1	(+)	1	M L Q F E K L Q Y S R L P Q K K L A K I S G G F N R V G Y N F G K S V R H V V D A I G S V A G I R G I L K S I R	56	Lactiplantibacillus plantar...
WP_062688856.1	(+)	1	M L Q F D K L Q Y S R L P Q K K L A K I S G G F N R G G Y N F G K S V R H V V D A I G S V A G I R G I L K S I R	56	Lactiplantibacillus plantar...
WP_102115620.1	(+)	1	M L Q F E K L Q Y S R L P Q K K L A K I S G G F N R G G Y N F G K S V R H V V D A I G S V A G I R G I L R S I R	56	Lactiplantibacillus plantar...
WP_070083242.1	(+)	1	M L Q F E K L Q Y S W L P Q K K L A K I S G G F N R G G Y N F G K S V R H V V D A I G S V A G I R G I L K S I R	56	Lactiplantibacillus plantar...
WP_064971884.1	(+)	1	M L Q F E K L Q Y S R L P Q K K L A K I S G S F N R G G Y N F G K S V R H V V D A I G S V A G I R G I L K S I R	56	Lactiplantibacillus plantar...
WP_181589728.1	(+)	1	M L Q F E K L Q Y S R L P Q K K L A K I S G G F N W G G Y N F G K S V R H V V D A I G S V A G I R G I L K S I R	56	Lactiplantibacillus plantar...
WP_128729524.1	(+)	1	M L Q F E K L Q Y S R L L Q K K L A K I S G G F N R G G Y N F G K S V R H V V D A I G S V A G I R G I L K S I R	56	Lactiplantibacillus plantar...
AHB33811.1	(+)	1	M L Q F E K L Q Y S R L P Q K K L A K I S G G F N R G G Y N F G K S V R H V V D A I G S V A G I R G I L - N I R G	56	Lactiplantibacillus plantar...
WP_122211246.1	(+)	1	M L K F D K L Q Y S N L S Q K K L T A V S G G F N R S G Y N F G K N V R H V C D A I S S A L G V R S V W K S I R	56	Lactobacillus pentosus
AIS39801.1	(+)	1	G G F N R G G Y N F G K S V R H V V D A I G S V A G I R G I L K S I R	35	Lactiplantibacillus plantar...
AGE45842.1	(+)	1	F N R G G Y N F G K S V R H V V D A I G S V A G I R G I L K S I R	33	Lactiplantibacillus plantar...
AGL63080.2	(+)	1	M L Q F E K L Q Y S R L P Q K K L A K I S G G F N R G G Y N F G K S V R H	37	Lactobacillus plantarum ...
QGV13665.1	(+)	1	F G K S V R H V V D A I G S V A G I R G I L K S I R	26	Lactiplantibacillus plantar...

NCBI Multiple Sequence Alignment Viewer, Version 1.18.0

Sequence ID	Start	Alignment	End	Organism
QHN60324.1 (+)	1	MKKFLVLRDRELNAISGGVFHAYSARGVRNNYKSAVGPADWVISAVRGFIHG	52	Lactiplantibacillus plantar...
WP_080440456.1 (+)	1	MKKFLVLRDRELNAISGGVFHAYSARGVRNNYKSAVGPADWVISAVRGFIHGYSSSHQV	59	Lactiplantibacillus plantar...
WP_003643811.1 (+)	1	MKKFLVLRDRELNAISGGVFHAYSARGVRNNYKSAVGPADWVISAVRGFIHG	52	Lactobacillaceae
WP_027822764.1 (+)	1	MKKFLVLRDRELNAISGGVFHAYSARGVRNNYKSAVGPADWVISAIRGFIHG	52	Lactiplantibacillus plantar...
WP_063852716.1 (+)	1	MKKFLVLRDRELNAVSGGVFHAYSARGVRNNYKSAVGPADWVISAVRGFIHG	52	Lactiplantibacillus plantar...
WP_021356667.1 (+)	1	MKKFLVLRDRELNSISGGVFHAYSARGVRNNYKSAVGPADWIISAVRGFIHG	52	Lactobacillaceae
WP_015825124.1 (+)	1	MKKFLVLSDRELNAISGGVFHAYSARGVRNNYKSAVGPADWVISAVRGFIHG	52	Lactiplantibacillus plantar...
QGV13666.1 (+)	1	MKKFLVLRDRELNAISCGVFHAYSARGVRNNYKSAVGPADWVISAVRGFIHG	52	Lactiplantibacillus plantar...
AFJ79568.1 (+)	1	MKKFLVLRDRELNSISGGVFHAYSARGVRNNYKSAVGPADWXISAVRGFIHG	52	Lactiplantibacillus plantar...
WP_070083241.1 (+)	1	MKKFLVLRDRELNAISGGVFHAYSARGVRNNYKSAVGPADWGISAVRGFIHG	52	Lactiplantibacillus plantar...
WP_033609517.1 (+)	1	MKKFLVLRGRELNAISGGVFHAYSARGVRNNYKSAVGPADWVISAVRGFIHG	52	Lactobacillus paraplantar...
WP_024521253.1 (+)	1	MKKFLVLRDRELXXISGGVFHAYSARGVRNNYKSAVGPADWIISAVRGFIHG	52	Lactiplantibacillus plantar...
WP_147781261.1 (+)	1	MKKFLVLRDRELNAISGGVFHAYSARGVRNNYKSAVGPADWVISAVRGF---ESVKYLV	56	Lactiplantibacillus plantar...
WP_122211247.1 (+)	1	MEKFLVLHDNELNTISGGVFHAYSARGVRNNYKSAVGPADWVISAVRGFIHG	52	Lactobacillus pentosus
WP_072534274.1 (+)	1	MKKFLVLRDRELNAISGGVFHAYSARGVRNNYKSAVGPADWVISAV	46	Lactiplantibacillus plantar...
WP_003641984.1 (+)	1	------MRDRELNAISGGVFHAYSARGVRNNYKSAVGPADWVISAVRGFIHG	46	Lactiplantibacillus plantar...
AGE45843.1 (+)	1	------------------VFHAYSARGVRNNYKSAVGPADWVISAVRGFIHG	34	Lactiplantibacillus plantar...
