# Supplementary material for: Enhancement of the antibacterial potential of plantaricin by incorporation into silver nanoparticles
Source: J Genet Eng Biotechnol. 2021 Jan 20;19:13. doi: 10.1186/s43141-020-00093-z (PMC7817718; doi:10.1186/s43141-020-00093-z)
Supplement: Supplementary file 4 — Additional file 4. Phyre Investigator output for plnE mature peptide with c2juiA template. Homology using Phyre2 of plnE peptide with template c2juiA and secondary structure prediction. [file 43141_2020_93_MOESM4_ESM.pdf]

[Return to main results](#) Retrieve Phyre Job Id Fetch

Job Description	pln_e____		
Confidence	99.80%	Date	Mon Sep 14 09:41:08 BST 2020
Rank	1	Aligned Residues	33
% Identity	97%	Template	c2juiA_
PDB info	**PDB header:**toxin	**Chain:** A: **PDB Molecule:**plne;	**PDBTitle:** three-dimensional structure of the two peptides that2 constitute the two-peptide bacteriocin plantaracin ef
Resolution	UNK		

Beta testing - Please contact [Lawrence Kelley](#) with problems/suggestions

Click one of the Quality or Function tabs below and press an analysis button.
A brief explanation of the analysis will appear here..

Click an analysis button. A key explaining the colours will appear here

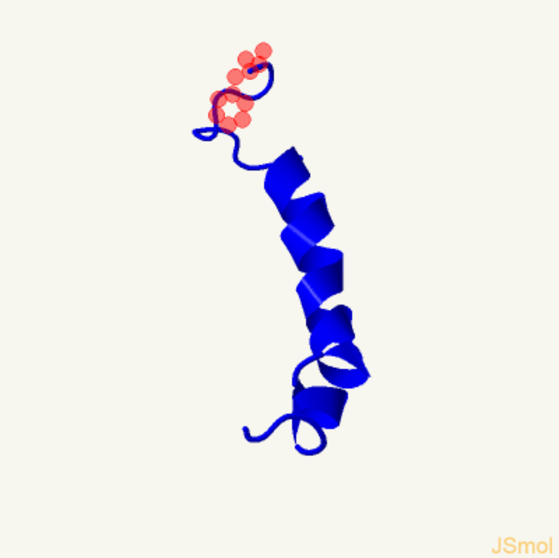

Analyses

Quality Function

ProQ2 quality assessment

Clashes

Rotamers

Ramachandran analysis

Alignment confidence

Disorder

Residue: PHE 1

Sequence profile **Mutations**

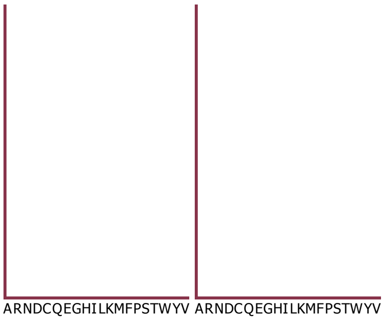

Take JMol snapshot | Show All analyses | Hide All analyses | Clear Selection

Hover over a residue below to see info. Click to spacefill.

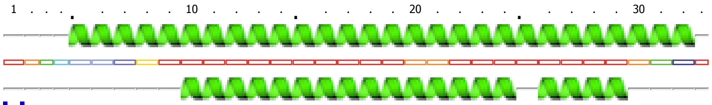

Predicted Secondary structure
SS Confidence
Model Secondary structure
Query Sequence FNRDGYNFGKSVRHVVDAIGSVAGIRGILKSIR
Modelled Residues FNRDGYNFGKSVRHVVDAIGSVAGIRGILKSIR

Please cite: The Phyre2 web portal for protein modeling, prediction and analysis
Kelley LA *et al. Nature Protocols* 10, 845-858 (2015)[paper][Citation link]
For mutational analysis, please cite: SuSPect: Enhanced Prediction of Single Amino Acid Variant (SAV) Phenotype Using Network Features
Yates *et al. J Mol Biol.* 426(14): 2692-2701 (2014)[paper][SuSPect Server]
