# Supplementary material for: Enhancement of the antibacterial potential of plantaricin by incorporation into silver nanoparticles
Source: J Genet Eng Biotechnol. 2021 Jan 20;19:13. doi: 10.1186/s43141-020-00093-z (PMC7817718; doi:10.1186/s43141-020-00093-z)
Supplement: Supplementary file 5 — Additional file 5. Phyre Investigator output for PlnF mature peptide with c2rlwA template. Homology using Phyre2 of PlnF peptide with template c2rlwA and secondary structure prediction. [file 43141_2020_93_MOESM5_ESM.pdf]

Return to main results

Retrieve Phyre Job Id [Fetch]

Investigator

Job Description	pln_f____		
Confidence	99.91%	Date	Mon Sep 14 10:47:43 BST 2020
Rank	1	Aligned Residues	34
% Identity	100%	Template	c2rlwA_
PDB info	**PDB header:**toxin	**Chain:** A: **PDB Molecule:**plnf;	**PDBTitle:** three-dimensional structure of the two peptides that2 constitute the two-peptide bacteriocin plantaracin ef
Resolution	UNK		

Beta testing - Please contact Lawrence Kelley with problems/suggestions

Click one of the Quality or Function tabs below and press an analysis button.
A brief explanation of the analysis will appear here..

Click an analysis button. A key explaining the colours will appear here

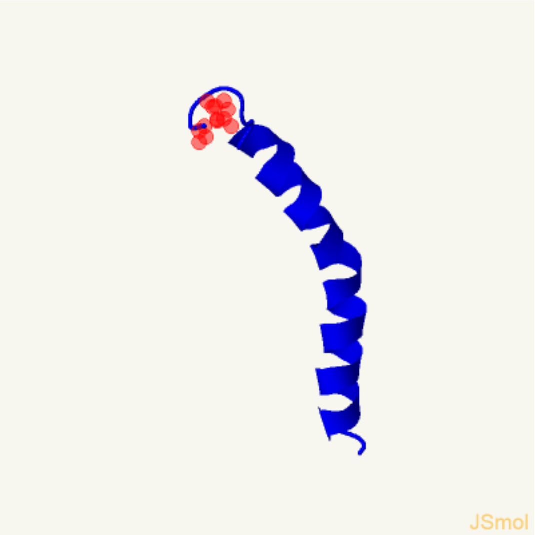

Analyses

Quality Function

- ProQ2 quality assessment
- Clashes
- Rotamers
- Ramachandran analysis
- Alignment confidence
- Disorder

Residue: VAL 1

Sequence profile

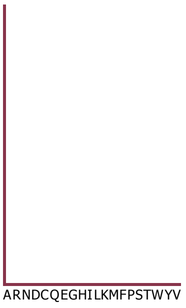

Mutations

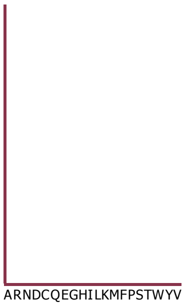

Take JMol snapshot | Show All analyses | Hide All analyses | Clear Selection

Hover over a residue below to see info. Click to spacefill.

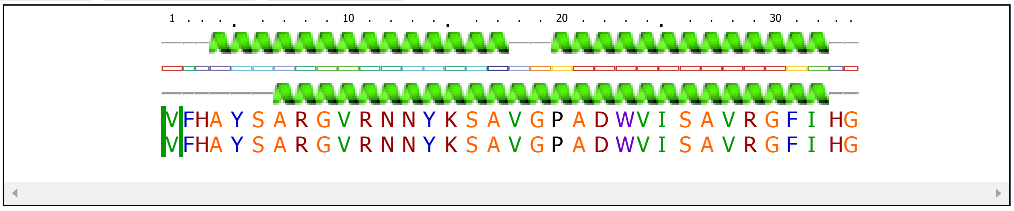

Please cite: The Phyre2 web portal for protein modeling, prediction and analysis
Kelley LA *et al. Nature Protocols* 10, 845-858 (2015)[paper][Citation link]
For mutational analysis, please cite: SuSPect: Enhanced Prediction of Single Amino Acid Variant (SAV) Phenotype Using Network Features
Yates *et al. J Mol Biol.* 426(14): 2692-2701 (2014)[paper][SuSPect Server]
