# Supplementary material for: Enhancement of the antibacterial potential of plantaricin by incorporation into silver nanoparticles
Source: J Genet Eng Biotechnol. 2021 Jan 20;19:13. doi: 10.1186/s43141-020-00093-z (PMC7817718; doi:10.1186/s43141-020-00093-z)
Supplement: Supplementary file 6 — Additional file 6. Physico-chemical properties and 3D-structure of peptides. Ccomputational software used to analyses physico-chemical properties and 3D-structure of mature peptides pln E and pln F. [file 43141_2020_93_MOESM6_ESM.pdf]

Physicochemical properties of putative mature pln EF

calculated molecular masses, net charge at pH 7, and grand average of hydropathicity (GRAVY) were predicted by using ProtParam (http://web.expasy.org/protparam/). The GRAVY value was calculated as the sum of hydropathy values of all amino acid residues in the peptide.

Pln E pepides

```
FNRDGYNFGKSVRHVVDAIGSVAGIRGILKSIR
cccccccccccceeeehhcccchhhhhhhhhhcc
Sequence length :    33
HNN :
   Alpha helix      (Hh) :    12 is  36.36%
   3₁₀ helix        (Gg) :     0 is   0.00%
   Pi helix         (Ii) :     0 is   0.00%
   Beta bridge      (Bb) :     0 is   0.00%
   Extended strand  (Ee) :     4 is  12.12%
   Beta turn        (Tt) :     0 is   0.00%
   Bend region      (Ss) :     0 is   0.00%
   Random coil      (Cc) :    17 is  51.52%
   Ambiguous states (?)  :     0 is   0.00%
   Other states          :     0 is   0.00%
```

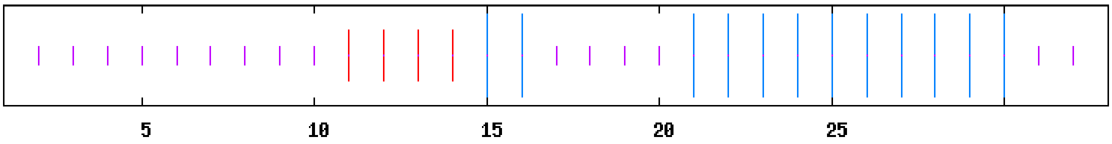

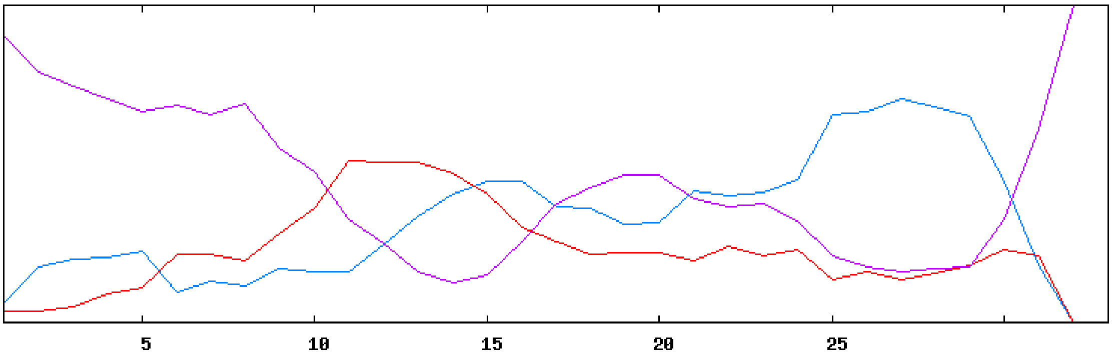

Prediction result file (text): [HNN]

Number of amino acids: 33

Molecular weight: 3603.15

Theoretical pI: 10.93

Amino acid composition: CSV format

Ala	(A)	2	6.1%
Arg	(R)	4	12.1%
Asn	(N)	2	6.1%
Asp	(D)	2	6.1%
Cys	(C)	0	0.0%
Gln	(Q)	0	0.0%
Glu	(E)	0	0.0%
Gly	(G)	5	15.2%
His	(H)	1	3.0%
Ile	(I)	4	12.1%
Leu	(L)	1	3.0%
Lys	(K)	2	6.1%
Met	(M)	0	0.0%
Phe	(F)	2	6.1%
Pro	(P)	0	0.0%
Ser	(S)	3	9.1%
Thr	(T)	0	0.0%
Trp	(W)	0	0.0%
Tyr	(Y)	1	3.0%
Val	(V)	4	12.1%
Pyl	(O)	0	0.0%
Sec	(U)	0	0.0%
	(B)	0	0.0%
	(Z)	0	0.0%
	(X)	0	0.0%

Total number of negatively charged residues (Asp + Glu): 2
Total number of positively charged residues (Arg + Lys): 6

Atomic composition:

Carbon	C	160
Hydrogen	H	261
Nitrogen	N	51
Oxygen	O	44
Sulfur	S	0

Formula: $C_{160}H_{261}N_{51}O_{44}$
Total number of atoms: 516

Extinction coefficients:

This protein does not contain any Trp residues. Experience shows that this could result in more than 10% error in the computed extinction coefficient.

Extinction coefficients are in units of M^{-1} cm^{-1}, at 280 nm measured in water.

Ext. coefficient	1490
Abs 0.1% (=1 g/l)	0.414

Estimated half-life:

The N-terminal of the sequence considered is F (Phe).

The estimated half-life is: 1.1 hours (mammalian reticulocytes, in vitro).
3 min (yeast, in vivo).
2 min (Escherichia coli, in vivo).

Instability index:

The instability index (II) is computed to be -3.18
This classifies the protein as stable.

Aliphatic index: 100.30

Grand average of hydropathicity (GRAVY): -0.027

Pln F peptides

```
        10        20        30
         |         |         |
VFHAYSARGVRNNYKSAVGPADWVISAVRGFIHG
ceeeeccccccccccccccchhhhhhhhhhhccc
```

Sequence length : 34

HNN :

Alpha helix	(Hh) :	11	is	32.35%
3_{10} helix	(Gg) :	0	is	0.00%
Pi helix	(Ii) :	0	is	0.00%
Beta bridge	(Bb) :	0	is	0.00%
Extended strand	(Ee) :	4	is	11.76%
Beta turn	(Tt) :	0	is	0.00%
Bend region	(Ss) :	0	is	0.00%
Random coil	(Cc) :	19	is	55.88%
Ambiguous states (?)	:	0	is	0.00%
Other states	:	0	is	0.00%

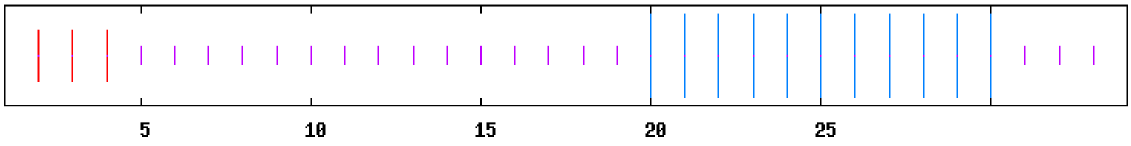

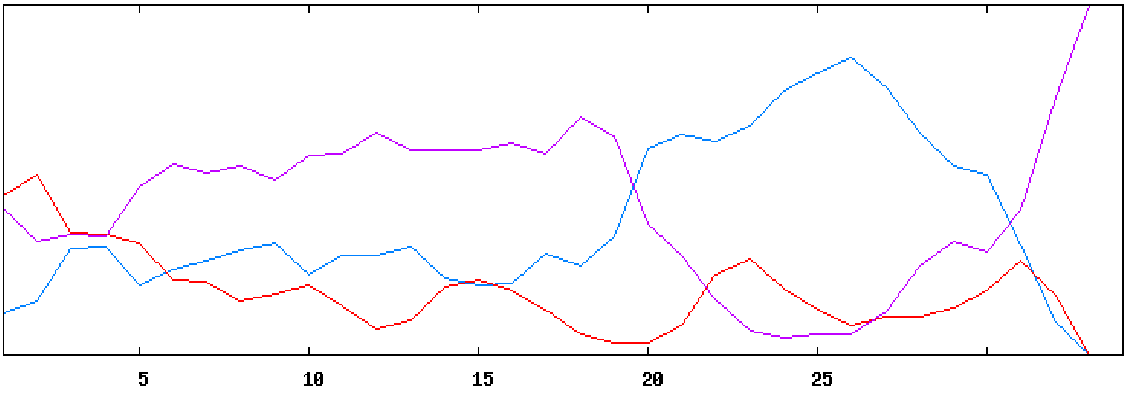

Prediction result file (text): [HNN]

User-provided sequence:

```
        10         20         30
VFHAYSARGV RNNYKSAVGP ADWVISAVRG FIHG
```

References and documentation are available.

Number of amino acids: 34

Molecular weight: 3703.18

Theoretical pI: 10.27

CSV format

Amino acid composition:

Ala	(A)	5	14.7%
Arg	(R)	3	8.8%
Asn	(N)	2	5.9%
Asp	(D)	1	2.9%
Cys	(C)	0	0.0%
Gln	(Q)	0	0.0%
Glu	(E)	0	0.0%
Gly	(G)	4	11.8%
His	(H)	2	5.9%
Ile	(I)	2	5.9%
Leu	(L)	0	0.0%
Lys	(K)	1	2.9%
Met	(M)	0	0.0%
Phe	(F)	2	5.9%
Pro	(P)	1	2.9%
Ser	(S)	3	8.8%
Thr	(T)	0	0.0%
Trp	(W)	1	2.9%
Tyr	(Y)	2	5.9%
Val	(V)	5	14.7%
Pyl	(O)	0	0.0%
Sec	(U)	0	0.0%

(B)	0	0.0%
(Z)	0	0.0%

(X)	0	0.0%

Total number of negatively charged residues (Asp + Glu): 1
Total number of positively charged residues (Arg + Lys): 4

Atomic composition:

Carbon	C	169
Hydrogen	H	253
Nitrogen	N	51
Oxygen	O	44
Sulfur	S	0

Formula: $C_{169}H_{253}N_{51}O_{44}$
Total number of atoms: 517

Extinction coefficients:

Extinction coefficients are in units of M^{-1} cm^{-1}, at 280 nm measured in water.

Ext. coefficient	8480
Abs 0.1% (=1 g/l)	2.290

Estimated half-life:

The N-terminal of the sequence considered is V (Val).

The estimated half-life is: 100 hours (mammalian reticulocytes, in vitro).
>20 hours (yeast, in vivo).
>10 hours (Escherichia coli, in vivo).

Instability index:

The instability index (II) is computed to be 7.09
This classifies the protein as stable.

Aliphatic index: 80.29

Grand average of hydropathicity (GRAVY): 0.035
